# Denosumab Versus Bisphosphonates in Glucocorticoid-Induced Osteoporosis: A Systematic Review

**DOI:** 10.7759/cureus.99602

**Published:** 2025-12-19

**Authors:** Anas E Ahmed, Waleed A Alzaylaee, Abdullah S Suhluli, Shahd A Essa, Latifah M Bahkali, Alanood M Hakami, Razan A Alshamrani, Fahad R Alharbi, Roaa H Alhazmi, Tassnim H Khurayzi, Abdulmalik K Almihbash

**Affiliations:** 1 Community Medicine, Jazan University, Jazan, SAU; 2 Internal Medicine, King Fahad Central Hospital, Jazan, SAU; 3 Medicine, Jazan University, Jazan, SAU; 4 Medicine, King Saud bin Abdulaziz University for Health Sciences, Riyadh, SAU; 5 Medicine, Tabuk University, Tabuk, SAU; 6 Pharmacy, Jazan University, Jazan, SAU; 7 Internal Medicine, Tabuk University, Tabuk, SAU

**Keywords:** bisphosphonates, bone microarchitecture, bone mineral density, bone turnover markers, denosumab, glucocorticoid-induced osteoporosis, glucocorticoid therapy

## Abstract

Glucocorticoid-induced osteoporosis (GIOP) is a major cause of secondary bone loss characterized by rapid trabecular deterioration and increased fracture risk, and while bisphosphonates are widely used as first-line therapy, the comparative effectiveness of denosumab remains clinically relevant. This systematic review synthesized evidence from randomized trials, imaging studies, and observational cohorts evaluating denosumab versus bisphosphonates in adults receiving glucocorticoids, focusing on bone mineral density, bone turnover, microarchitecture, fractures, and safety. Denosumab consistently produced greater increases in lumbar spine bone mineral density, with modest but generally favorable effects at the hip and variable advantages at the femoral neck. High-resolution imaging suggested superior preservation of cortical thickness, trabecular density, and estimated bone strength with denosumab, accompanied by deeper suppression of bone turnover markers across studies. Fracture outcomes did not demonstrate meaningful differences between treatments, likely due to limited power and relatively short follow-up. Safety profiles were broadly comparable, without significant variation in adverse events or serious complications. Overall, denosumab appears to offer stronger densitometric and mechanistic benefits than bisphosphonates in GIOP, supporting its use in patients who are inadequately responsive to alternative therapies or require long-term glucocorticoid treatment, although larger and longer trials with fracture endpoints are still needed.

## Introduction and background

Glucocorticoid-induced osteoporosis (GIOP) is the most common form of secondary osteoporosis and a major concern for patients requiring long-term systemic glucocorticoid therapy. Even low doses accelerate bone loss by disrupting normal bone remodeling, leading to reduced bone formation, increased bone resorption, and a rapid decline in bone mineral density (BMD) within the first few months of treatment [1-3]. These changes significantly increase the risk of fragility fractures, particularly vertebral fractures, which contribute to substantial morbidity and healthcare burden. Despite multiple available therapeutic options, optimizing bone protection in glucocorticoid-treated patients remains a clinical priority [1-4].

Bisphosphonates have traditionally served as first-line therapy due to their established ability to reduce bone turnover and increase BMD at major skeletal sites [2,5,6]. However, limitations such as dependence on remodeling surfaces, poor gastrointestinal absorption, long skeletal retention, and reduced effectiveness in some high-risk or previously treated patients have highlighted the need for alternatives [2,5,6].

Denosumab, a monoclonal antibody targeting receptor activator of nuclear factor κB ligand (RANKL), offers a distinct antiresorptive mechanism by suppressing osteoclast formation and activity. It produces rapid reductions in bone turnover and substantial gains in both cortical and trabecular bone across various osteoporosis populations [1,2,3]. Its reversible action, convenient administration, and suitability for patients with renal impairment further enhance its clinical utility [1,4,6]. Emerging evidence suggests that denosumab may provide greater improvements in BMD than bisphosphonates in individuals receiving glucocorticoids [1-3,6].

However, uncertainty persists regarding the comparative benefits and risks of denosumab, specifically in the context of GIOP. Existing studies vary in patient characteristics, glucocorticoid exposure, duration, and outcome reporting, and long-term fracture data remain limited [1-6]. A systematic evaluation of the available evidence is therefore warranted to clarify whether denosumab provides meaningful advantages over bisphosphonates for patients undergoing glucocorticoid therapy [1-6].

This systematic review aims to synthesize evidence comparing denosumab and bisphosphonates in adults with GIOP, focusing on changes in BMD at key skeletal sites, bone turnover markers, fractures, and adverse events to inform clinical decision making in this high-risk population [1-6].

## Review

Methods

Literature Search Strategy

A systematic search was conducted across PubMed, Scopus, Web of Science, and the Cochrane Library for all available studies up to December 2025. Search terms included combinations related to glucocorticoid-induced osteoporosis, osteoporosis and bone density outcomes, denosumab, and bisphosphonates, with filters for comparative study designs. Searches were restricted to human studies in English and to original research directly comparing denosumab with bisphosphonates in adults receiving systemic glucocorticoids.

Eligibility Criteria

Eligibility was defined according to the Population-Intervention-Comparator-Outcome framework [7]. Included studies examined adults receiving systemic glucocorticoids for any medical condition, with denosumab as the intervention and any oral or intravenous bisphosphonate as the comparator. Studies were required to report at least one skeletal outcome, including BMD, bone turnover markers, bone microarchitecture, or fractures. Eligible designs included randomized controlled trials (RCTs), prospective studies, and retrospective comparative cohorts. Exclusion criteria were non-comparative designs, non-bisphosphonate comparators, case reports, reviews, conference abstracts, pediatric studies, laboratory research, and studies lacking extractable skeletal outcomes.

Study Selection

All records were imported into reference management software for duplicate removal. Two reviewers independently screened titles and abstracts, followed by full-text assessment of potentially eligible studies. Disagreements were resolved through discussion or adjudication by a third reviewer. The selection process is summarized in a Preferred Reporting Items for Systematic Reviews and Meta-Analyses (PRISMA) flow diagram [8].

Data Extraction and Quality Appraisal

Two reviewers independently extracted data on study design, participant characteristics, glucocorticoid exposure, interventions, comparators, follow-up duration, and skeletal outcomes. Extracted data were cross-checked for accuracy. Quality assessment was performed using the Downs and Black checklist, evaluating reporting quality, external validity, internal validity, and statistical power [9]. Quality ratings informed the interpretation of findings in the qualitative synthesis.

Results

Study Selection

**Figure 1:**
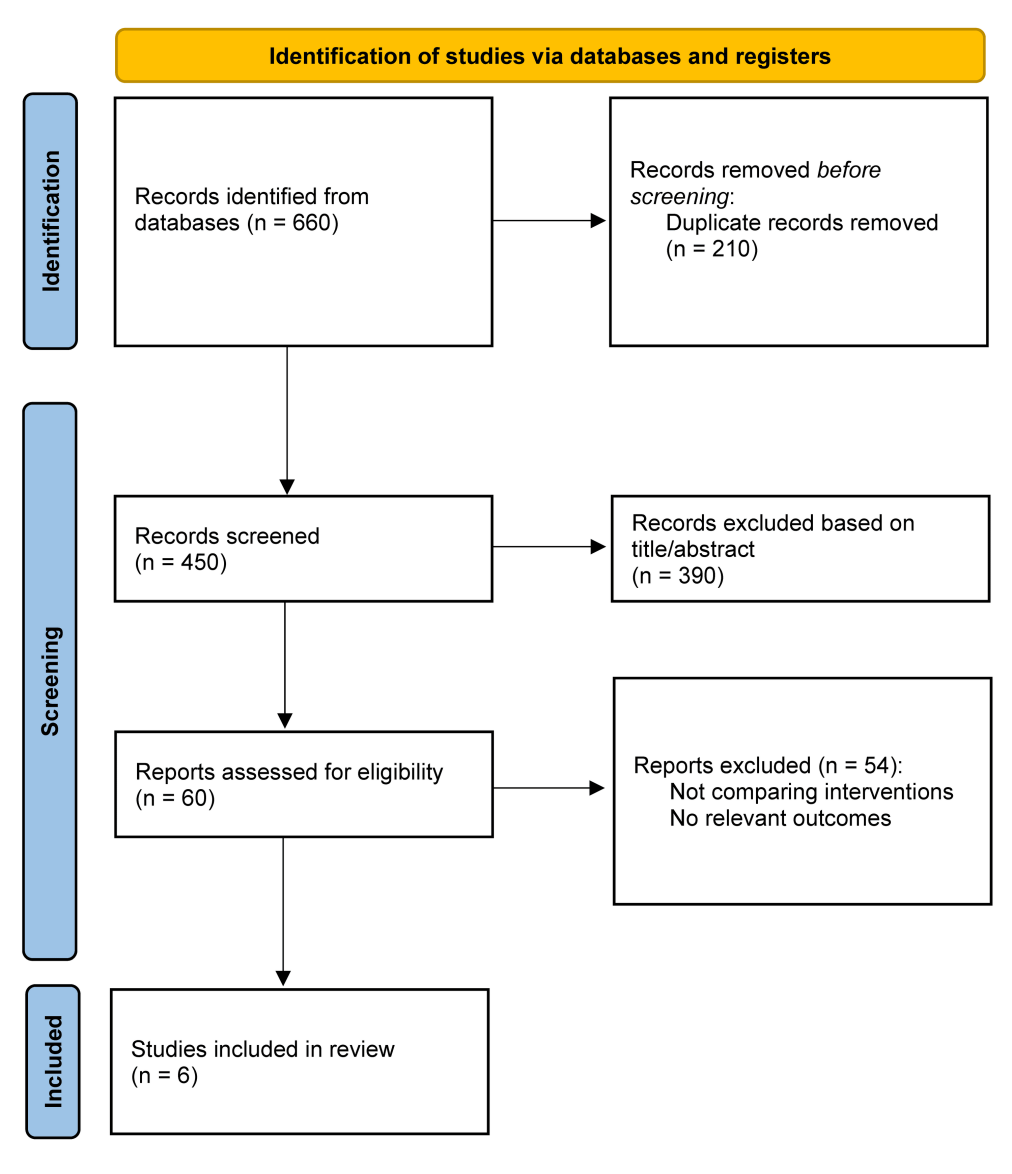
PRISMA flow diagram of the study selection process PRISMA - Preferred Reporting Items for Systematic Reviews and Meta-Analyses

The database search initially identified 660 records. After removing duplicates, 450 studies were screened, and 390 were excluded based on title and abstract. Sixty full-text articles were assessed, of which 54 were excluded for lacking comparative interventions, not involving glucocorticoid-induced osteoporosis (GIOP) populations, or not reporting relevant skeletal outcomes. Six studies met all inclusion criteria [1-6]. Heterogeneity in study design and outcomes precluded quantitative meta-analysis (Figure 1).

Baseline Characteristics of Included Studies

The six included studies consisted of four randomized controlled trials (RCTs), one high-resolution peripheral quantitative computed tomography (HR-pQCT) mechanistic substudy, and one long-term retrospective cohort. The largest RCT by Saag et al. [3] demonstrated greater improvements in lumbar spine, total hip, and femoral neck bone mineral density (BMD) with denosumab compared with risedronate. The HR-pQCT substudy by Geusens et al. [1] provided mechanistic evidence of superior preservation of cortical thickness, trabecular density, and estimated bone strength with denosumab. Additional RCTs by Mok et al. [2,4] and Iseri et al. [5] reported larger lumbar spine BMD gains and deeper suppression of bone turnover markers with denosumab. The retrospective cohort by Handa et al. [6] demonstrated sustained long-term increases in lumbar spine and hip BMD with denosumab, whereas patients treated with bisphosphonates exhibited minimal or declining trends over time (Table 1).

**Table 1:** Summary of characteristics of the included studies Summary of randomized controlled trials and observational studies comparing denosumab with bisphosphonates in glucocorticoid-induced osteoporosis (GIOP). AE - adverse event; ALN - alendronate; BAP - bone-specific alkaline phosphatase; BMD - bone mineral density; BP - bisphosphonate; BTM - bone turnover marker; CIDP - chronic inflammatory demyelinating polyneuropathy; CTX - C-terminal telopeptide of type I collagen; D - denosumab; FN - femoral neck; GC - glucocorticoid; GIOP - glucocorticoid-induced osteoporosis; HR-pQCT - high-resolution peripheral quantitative computed tomography; IBN - ibandronate; LS - lumbar spine; MG - myasthenia gravis; NMOSD -neuromyelitis optica spectrum disorder; NS - not significant; ONJ - osteonecrosis of the jaw; P1NP - procollagen type I N-terminal propeptide; PF - prednisone/prednisolone (PSL); RIS - risedronate; RCT - randomized controlled trial; SAE - serious adverse event; SC - subcutaneous; SLE - systemic lupus erythematosus; TH - total hip; TRACP-5b - tartrate-resistant acid phosphatase 5b; UD - ultradistal; URTI - upper respiratory tract infection

Study	Country	Study design	Population	Glucocorticoid details	Sample size (denosumab vs BP)	Denosumab regimen	Bisphosphonate regimen	Follow-up duration	Primary outcome	Secondary outcomes	Lumbar spine BMD change (%)	Total hip BMD change (%)	Femoral neck BMD change (%)	Bone turnover markers	Fractures	Adverse events	Key notes
Geusens et al. [1]	Multinational	RCT substudy (HR-pQCT imaging)	GIOP patients (initiators & continuers)	Prednisone ≥7.5 mg/day	D: 55, BP: 55	60 mg SC q6 months	Risedronate 5 mg daily	24 months	Bone strength (failure load, radius & tibia)	Cortical & trabecular microarchitecture	Not measured	Not measured	Not measured	Not measured	Not assessed	No new safety signals	Denosumab preserved/improved bone strength; BP associated with cortical deterioration
Mok et al. [2]	Hong Kong	Randomized controlled trial (open-label, 1:1)	Women on ≥2 yrs of oral BP + long-term GCs	Prednisolone ≥2.5 mg/day for ≥1 yr (mean 101 mo; mean dose 4.4 mg/day)	D: 21, BP: 21	60 mg SC q6 months	Continued BP: ALN 79%, RIS 12%, IBN 10%	12 months	Lumbar spine BMD change	Hip BMD; FN BMD; BTMs; fractures; safety	+3.4% ± 0.9% vs +1.5% ± 0.4% (p = 0.01 adj.)	+1.38% vs +0.80% (NS)	−0.14% vs +0.57% (NS)	Denosumab suppressed P1NP & β-CTX more strongly	No vertebral or non-vertebral fractures	URTI slightly higher in D; no SAEs	BP inadequate responders; denosumab superior for LS BMD & BTMs
Saag et al. [3]	Multinational	Randomized, double-blind, double-dummy Phase III RCT	Adults with GIOP (initiators & continuers)	≥7.5 mg/day prednisone	D: 398, BP: 397	60 mg SC q6 months	Risedronate 5 mg daily	24 months	Lumbar spine BMD change	TH & FN BMD; radius BMD; BTMs; fractures; safety	Initiators: +6.2% vs +1.7%; Continuers: +6.4% vs +3.2%	Initiators: +3.1% vs 0.0%; Continuers: +2.9% vs +0.5%	Initiators: +1.5% vs −0.9%; Continuers: +2.2% vs +0.4%	Stronger CTX & P1NP suppression with denosumab	Any: 8.8% vs 9.1%; Vertebral: 4.4% vs 6.9%; Non-vertebral: 5.3% vs 3.8%	Similar AE rates; 1 atypical femoral fracture in D; no ONJ	Largest high-quality RCT; robust evidence of denosumab superiority
Handa et al. [4]	Japan	Retrospective cohort	Neuroimmunological disorders (NMOSD, MG, CIDP) on long-term GCs	Median PSL ~10 mg/day; >3 months	D: 23, BP: 34	60 mg SC q6 months	Mixed BP: ALN, RIS, minodronate	Up to 6 years	LS & TH BMD % change	Fractures	LS: +4.1 → +9.7 → +6.6% vs BP −0.8 → −0.6 → −0.7%	TH: +2.3 → +4.3 → +7.1% vs BP −2.1 → −2.2 → −2.6%	Not reported	Not measured	2/23 vs 2/34 (2 yrs); 2/8 vs 5/33 (6 yrs)	No hypocalcemia, no ONJ; similar AEs	Longest follow-up; denosumab consistently superior; retrospective design limits certainty
Mok et al. [5]	Hong Kong	Randomized controlled trial (open-label, 1:1)	Adults on long-term GCs (81% SLE)	Prednisolone ≥2.5 mg/day for ≥1 yr (mean 5.1 mg/day)	D: 69, ALN: 70	60 mg SC q6 months	Alendronate 70 mg weekly	12 months	Lumbar spine BMD	TH BMD; FN BMD; BTMs; AEs	+3.5% ± 2.5% vs +2.5% ± 2.9% (p = 0.045 adj.)	+0.9% vs +1.6% (NS)	+1.04% vs +1.5% (NS)	Stronger suppression: P1NP 53% vs 22%; CTX 57% vs 5.3%	2 vertebral fractures in each group	AE rates similar; no ONJ; mild infections slightly ↑ in D	Largest ALN comparison in GC users; denosumab superior for LS BMD & BTMs
Iseri et al. [6]	Japan	Prospective, randomized, open-label trial	Adults with glomerular disease & new GIOP	Median PSL 5 mg/day (29% <3 mo; 71% ≥3 mo)	D: 14, ALN: 14	60 mg SC q6 months + calcitriol	Alendronate 35 mg weekly + calcitriol	12 months	% change LS BMD	FN BMD; UD radius; BTMs; safety	+5.3% ± 1.0% (p<0.01); superior to ALN (p<0.05)	No significant change	+1.8% ± 1.1% (NS); ALN slightly declined	Strong suppression of TRACP-5b, BAP, t-P1NP	1 femoral neck fracture in D	Mild hypocalcemia; eczema flare; TB case; none severe	First ALN-controlled RCT in GIOP; denosumab significantly improved LS BMD

Quality Assessment

Overall quality was high. Saag et al. [3] achieved the highest score, and the RCTs by Mok et al. [2,4] and Iseri et al. [5] were rated good to excellent. The HR-pQCT substudy [1] demonstrated good internal validity despite a smaller imaging subset. The retrospective cohort [6] was rated Fair due to inherent design limitations but contributed meaningful long-term data (Table 2).

**Table 2:** Summary of the quality appraisal of the included studies Risk of bias assessment of studies evaluating denosumab versus bisphosphonates in glucocorticoid-induced osteoporosis (GIOP) using the Downs and Black checklist [9].

Study	Reporting (0-10)	External validity (0-3)	Internal validity - bias (0-7)	Internal validity - confounding (0-6)	Power (0-2)	Total score (0-28)	Quality rating
Geusens et al. [1]	10	1	7	5	0	23 / 28	Good
Mok et al. [2]	10	2	6	5	2	25 / 28	Good
Saag et al. [3]	10	3	7	6	2	28 / 28	Excellent
Handa et al. [4]	9	1	4	2	0	16 / 25	Fair
Mok et al. [5]	10	2	6	5	2	25 / 28	Good
Iseri et al. [6]	10	2	6	4	2	24 / 28	Good

Lumbar Spine BMD

Denosumab consistently produced the largest and most consistent gains in lumbar spine BMD. In the largest RCT [3], increases of +6.2% to +6.4% over 24 months exceeded those observed with risedronate. Similar superiority was reported in the RCTs by Mok et al. [2,4], in the trial by Iseri et al. [5], and in the long-term follow-up of Handa et al. [6].

Total Hip BMD

Total hip BMD generally favored denosumab. Saag et al. [3] reported significantly greater gains across all glucocorticoid exposure groups. Other RCTs reported smaller or nonsignificant differences [2,4,5], whereas the retrospective cohort demonstrated sustained improvements with denosumab and declines with bisphosphonates [6].

Femoral Neck BMD

Femoral neck outcomes were more variable. Saag et al. [3] observed significant improvements with denosumab, while smaller RCTs showed minimal between-group differences [2,4,5].

Bone Microarchitecture

The HR-pQCT substudy [1] demonstrated that denosumab more effectively preserved cortical thickness, trabecular density, and estimated bone strength at the radius and tibia, whereas risedronate was associated with cortical deterioration.

Bone Turnover Markers

All RCTs [2-5] consistently reported deeper suppression of bone turnover markers, including C-terminal telopeptide (CTX), procollagen type I N-terminal propeptide (P1NP), and tartrate-resistant acid phosphatase 5b (TRACP-5b), reflecting denosumab's potent antiresorptive activity.

Fracture Outcomes

Fracture rates were low across the studies. The largest RCT [3] and the long-term retrospective cohort [6] reported no significant differences in vertebral or nonvertebral fractures between the denosumab and bisphosphonate groups. Smaller RCTs reported no new fractures, limiting conclusions regarding comparative fracture reduction.

Safety Outcomes

Both therapies were generally well tolerated. Denosumab was associated with slightly higher rates of mild infections in some studies [2,4] and occasional hypocalcemia [5], whereas bisphosphonates were more frequently linked to gastrointestinal adverse effects. No cases of osteonecrosis of the jaw were reported. Overall, denosumab demonstrated consistent advantages in BMD and bone turnover suppression with comparable safety to bisphosphonates.

## Conclusions

This systematic review indicates that denosumab provides meaningful benefits over bisphosphonates for glucocorticoid-induced osteoporosis, particularly in improving lumbar spine BMD and suppressing bone turnover. While evidence for fracture reduction remains limited due to low event rates and short follow-up durations, the available data support denosumab as an effective therapeutic option, especially for patients who respond inadequately to bisphosphonates. Larger and longer-term RCTs with fracture endpoints are needed to better define its comparative effectiveness.
